# Synthesis of Hybrid Carbon Materials Consisting of N-Doped Microporous Carbon and Amorphous Carbon Nanotubes

**DOI:** 10.3390/ma13132997

**Published:** 2020-07-06

**Authors:** Wojciech Zielinski, Piotr Kamedulski, Aleksander Smolarkiewicz-Wyczachowski, Malgorzata Skorupska, Jerzy P. Lukaszewicz, Anna Ilnicka

**Affiliations:** 1 Faculty of Chemistry, Nicolaus Copernicus University in Torun, Gagarina 7, 87-100 Torun, Poland; wzielinski@umk.pl (W.Z.); pkamedulski@umk.pl (P.K.); asmolarkiewicz@gmail.com (A.S.-W.); m.skorupska@doktorant.umk.pl (M.S.); jerzy_lukaszewicz@o2.pl (J.P.L.); 2 Centre for Modern Interdisciplinary Technologies, Nicolaus Copernicus University in Torun, Wilenska 4, 87-100 Torun, Poland

**Keywords:** hybrid material, N-doped carbon, chitosan, amorphous carbon nanotubes, pyrrolic-N, quaternary-N, porous structure

## Abstract

The N-doped hybrid carbon materials containing amorphous carbon nanotubes (ACNTs) were obtained by free growth of a polymer at 200 °C. The improvement of electrical conductivity was achieved by a final carbonization at 600–800 °C under the flow of nitrogen. The microstructure of ACNT/N-doped hybrids was characterized using a transmission electron microscope and X-ray diffusion. Furthermore, their elemental composition was measured using energy-dispersive X-ray spectroscopy and an elemental analyzer. The experimental results indicated that the ACNTs had a diameter in the range of 40–60 nm and the N-doped carbon background contained nitrogen atoms in most bonded pyrrolic-N and quaternary-N groups. The results revealed that the microstructure of the as-grown nanotubes, prepared by the proposed method, is mainly amorphous. This technique introduces the advantages of low cost and process simplicity, which may redeem some drawbacks of the methods commonly used in ACNT synthesis.

## 1. Introduction

In the past two decades, the interest in carbon nanotubes (CNTs) has strongly increased. Generally, carbon nanotubes can be produced by different methods and the following are very popular: chemical vapor deposition (CVD) [1], laser ablation [2]. and the arc method [3]. Carbon nanotubes, with the nanometer size and tube-like shape, have different graphitization feature from the bulk carbon. Ci et al. investigated graphitization behavior of carbon nanotubes, produced by the floating catalyst method [4]. Recently, amorphous carbon nanotubes (ACNTs) have become the focus of research because their synthesis process is more facile than for carbon nanotubes. Firstly, ACNTs are produced at a lower temperature, with a large yield of production [5,6,7,8]. The walls of ACNTs are composed of many carbon clusters featuring long-distance disorder and short-distance order. In amorphous carbon nanotubes sp^2^ and sp^3^ bonds occur [9]. In general, amorphous carbon material contains a highly disordered network of carbon atoms that in more than 10% are sp^3^ bonds, but predominantly sp^2^ bonds [10]. The ACNTs have many unique structures, such as the amorphous wall, larger interior, as well as the nano-scale tubular shape and diameters [11,12,13]. Another type of carbon material with sp^2^ and sp^3^ bonds is the amorphous carbon nanowire (CNW) produced at 1200 °C by heating a pressed tablet of graphite powder mixed with nickel in a quartz tube [14]. Huang et al. reported that the CNWs form under high-bias-caused Joule heating with a diameter of about 10 nm and length of about 90 nm [15].

So far, various methods have been developed to produce ACNTs. It has been reported than ACNTs can be synthesized by a different method. Chen et al. prepared ACNTs using polyacrylonitrile and polystyrene as carbon precursors. The wall thickness of ACNTs was changed by using different concentrations of the precursor solution [16]. Hu et al. prepared ACNTs using glucose as a precursor [17]. Nishino et al. produced ACNTs from poly(tetrafluoroethylene) and ferrous chloride by the CVD method [18]. The synthesis of ACNTs proposed by Zhao et al. was based on the arc discharge method [19]. Suzuki et al. reported an advanced carbon coating method of forming amorphous carbon tubes through the reduction of the central copper oxide whisker [20]. Zhao et al. in situ formed ACNTs during the preparation of SnO_2_ nanowires [21]. Hu et al. synthesized ACNTs by pyrolysis of ferrocene confined in the nanopores of the anodic alumina membrane [22]. Im et al. reported the development of a CNTs/MnO_2_/graphene oxide ternary composite electrode and studied its electrochemical application [23]. Sarker et al. applied the anodic aluminum oxide template method and the ACNTs-MnO_2_ hybrids were further coated with graphene oxide [24] or composite ACNTs-MnO_2_ without graphene [25]. Depending on the applications, composites like ACNTs-ZnO [26], ACNTs-CuPc [7], ACNTs-NiO [27], ACNTs-TiO_2_ [28], ACNTs-Fe_2_O_3_-Mn_2_O_3_ [29], ACNTs-CuO [30], ACNTs-SiO_2_ [31], and ACNTs-polyvinyl chloride [32] were also used.

According to the available literature, hybrid and composite carbon materials have interesting electrochemical properties which are covered by a wide range of research [33]. Pahari et al. created amorphous carbon nanotubes in gram scale production and the incorporation of copper oxide significantly enhanced the electrochemical performance of supercapacitors [34]. Xu et al. and Liang et al. reported NiCoO_2_ nanosheets and SnO_2_ layer heterostructure supported by amorphous carbon nanotubes as an electrode for lithium storage properties [31,35]. In the same type of battery, Zhou et al. observed Sn nanoparticles in amorphous carbon nanotubes for enhanced lithium storage properties [36]. Also, Zhao et al. used amorphous carbon nanotubes as anode materials for lithium ion battery [37]. Numerous defects and large interlayer space are conducive to rapid diffusion and reversible storage for Li^+^ [38,39,40]. Xu et al. proposed using MoS_2_ nanosheets grown on amorphous carbon nanotubes as anode materials for sodium ion batteries [41]. Another field of application of ACNTs is adsorption of hazardous compounds. According to Bhowmik et al., ACNTs are a good sorbent for removing phenolic derivative compound, resorcinol and highly toxic arsenic (III) from water [42]. In addition, ACNTs adsorbents can be used to remove carcinogenic dyes, such as Rhodamine B, methyl orange, and Congo Red from an aqueous solution due to the presence of defects at the amorphous wall, more dangling bonds, and larger surface area as compared to sp^2^ graphite [43,44]. The defect site on the wall of the carbon network enables ACNTs to be used as sensors, a gaseous adsorbent, and catalyst supports [45]. In the light of the above review on ACNT-based hybrids, it can be concluded that none of the studies discovered how to combine the presence of the tubes with an increased level of nitrogen. Nitrogen-doped carbon matrixes are generally considered a non-metal electrocatalyst for Zn-air batteries [46] or an efficient electrode for a supercapacitor [47,48]. 

This paper aims to demonstrate the mold-free synthesis of ACNTs from a polymer through thermal transformation. The growth is combined with a parallel synthesis of a carbonaceous background intensively enriched with nitrogen. The N-doped background originates from a biopolymer, namely, chitosan which was intensively tested as a precursor for N-doped activated carbons. The synthesis will be optimized regarding the processing temperature and its duration as well as the mixing ratio of the main components.

## 2. Materials and Methods 

### 2.1. Preparation of ACNT/N-Doped AC without or with FeCl_3_

N-doped carbon, which was synthesized from chitosan following the previously described method, was used as a background for growth of ACNTs [49]. The carbon samples selected for these tests, which were produced by treatment with phosphoric(V) acid at concentrations of 2.46 and 5.53 mole dm^−3^ were labeled in the above cited paper as CH_2.46 and CH_5.53, respectively. These two samples, i.e., CH_2.46 and CH_5.53, after carbonization, but before removing the activator are described in this paper as C1 and C2, respectively. In this study, we used these samples. The 0.3 g or 0.4 g of N-doped carbon from chitosan was treated with furfuryl alcohol (FA) and phosphoric(V) acid (H_3_PO_4_) in 6:1 *v*/*v* ratio. Then, the mass was kept at 200 °C in an electric oven for 1 h. In the next step, the mass was carbonized at a high temperature in the range of 600–800 °C. The heating rate was 10 °C min^−1^ under the flow of N_2_ and hold time at max temperature was 1 h. After that, all carbons were thoroughly washed with distilled hot water (in an ultrasonic bath at 80 °C for 2 h) and next, in hot water under vacuum until the final pH equal to 7 was attained in the residual liquid. The test reaction with ammonium molybdate also checked for the absence of phosphate ions in the filtrate. The samples were then dried overnight in an electric oven. The obtained ACNT/N-doped AC hybrid carbon samples are designated as C1-T and C2-T, where C1 and C2 are CH_2.46 and CH_5.53, respectively (Table 1).

Additionally, in C1-T and C2-T series, anhydrous FeCl_3_ (0.01 g) was added to FA. The obtained mass with furfuryl alcohol, FeCl_3_, and H_3_PO_4_ was put into the heat oven at 200 °C for 1 h. Then the obtained sample was carbonized in a nitrogen atmosphere at 600–800 °C. The heating rate was 10 °C min^−1^ under the flow of N_2_ and hold time at max temperature was 1 h. Rinsing with water was performed in the same manner as described for C1-T and C2-T. The obtained ACNT/N-doped AC hybrid carbon samples are designated as C1-A-T and C2-A-T, where C1 and C2 are CH_2.46 and CH_5.53 N-doped carbon, respectively. A refers to the use of FeCl_3_ in the synthesis and T (6, 7, or 8) refers to the carbonization temperature of 600, 700, and 800 °C, respectively (Table 1).

### 2.2. Characterization Methods

The morphology of the samples was determined using a scanning electron microscope (SEM 1430 VP, LEO Electron Microscopy Ltd., Oberkochen, Germany) operating at 30 kV. The atomic structure of the samples was observed by a high-resolution transmission electron microscope (HRTEM FEI Tecnai F20 X-Twin, Brno, Czech Republic) with energy dispersive X-ray spectrometer (EDX) at an accelerating voltage of 200 kV. The porous structure of the samples was analyzed by a nitrogen adsorption experiment at 77 K using an automatic adsorption instrument, ASAP 2020 Plus (Micromeritics, Norcross, the United States of America). Before the analysis, carbon materials were outgassed in vacuum at 200 °C for 24 h. The surface areas and pore size distributions of the samples were calculated using the Brunauer–Emmett–Teller (BET) equation and the nonlocalized density functional theory (NLDFT) method, respectively. The elemental composition of the materials was analyzed by means of a combustion elemental analyzer (Vario CHN, Elementar Analysensysteme GmbH, Langenselbold, Germany). The X-ray photoelectron spectroscopy (XPS, PHI5000 VersaProbe II Scanning XPS Microprobe, Chigasaki, Japan) measurements were performed using a monochromatic Al Kα X-ray source. Survey spectra were recorded for all samples in the energy range of 0 to 1300 eV with a 0.5 eV step, high-resolution spectra were recorded with a 0.1 eV step. Raman spectra were obtained by a Renishaw InVia Raman analyzer (laser wavelength 532 nm, Renishaw Company, Gloucestershire, UK). X-ray diffraction (XRD) patterns of the samples were obtained with an X-ray diffractometer (X’Pert-Pro, Philips, Cambridge, the United Kingdom) equipped with a Cu Kα source at 40 kV and 30 mA.

## 3. Results and Discussion

**Figure 1:**
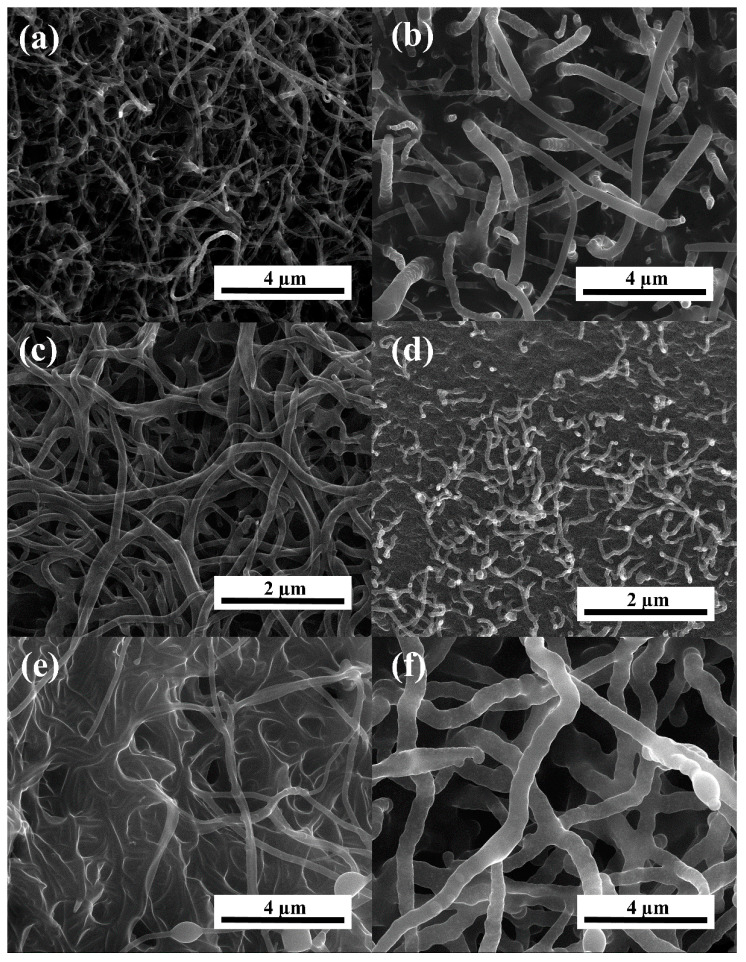
Scanning electron microscope (SEM) images for samples: (**a**) C1-6; (**b**) C1-A-6; (**c**) C1-7; (**d**) C1-8; (**e**) C2-6; and (**f**) C2-A-6.

The SEM analysis of (Figure 1) images delivered a direct proof of ACNTs formation on the surface of the activated carbon background. All images depict the growth of ACNTs using the direct thermal conversion of polyfurfuryl alcohol (PFA). The amorphous carbon nanotubes densely covered the background, leaving very limited access to the pristine background surface. Figure 1 shows that the surface of the carbonaceous nano-material is covered by randomly oriented tube-like objects. The SEM does not prove whether the tubular objects are crystalline or amorphous. In most cases, the tube tips are flat and rounded. Flat tip planes are perpendicular to the tubes’ axis. The average outer diameter of the ACNTs-AC samples depends on the type of carbon background (C1 and C2), carbonization temperature (600, 700, or 800 °C), and the use of additional catalyst (with or without FeCl_3_). The hybrid carbon materials obtained in the C1-T series for C1-6, C1-7, and C1-8 sample contain ACNTs with outer diameter of 43–76 nm, 53–153 nm, and 39–58 nm, respectively. The outer diameter of ACNTs for the C1-A-6, C1-A-7, and C1-A-8 samples is 85–170 nm, 83–116 nm, and 43–54 nm, respectively.

**Figure 2:**
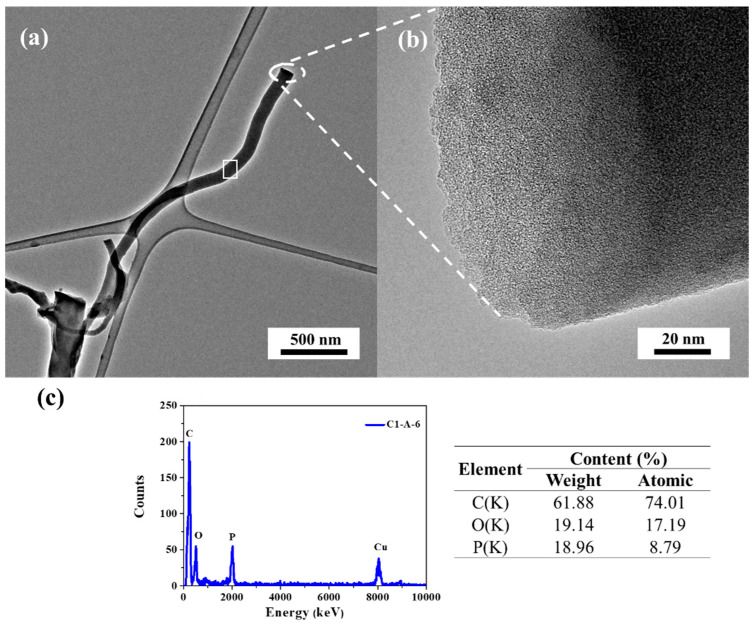
(**a**,**b**) High-resolution transmission electron microscope (HRTEM) images of a C1-A-6 sample with different magnifications and (**c**) energy dispersive X-ray spectrometer (EDX) elemental profiles and composition.

These results indicated that for the C1-A-T series obtained at an increasing carbonization temperature it is distinctive because of the minimized outer diameter of the tubes. For the C2-6 and C2-A-6 samples, the outer diameter is 145–219 and 150–170 nm, respectively. The C2 background used for ACNTs growth (at 600 °C) lead to ACNTs with larger outer diameter than for the C1 background (C1-6 and C1-A-6). The HRTEM images of (Figure 2a,b) indicated that the tubes are smooth and long, and the diameters range between 85–170 nm. Poor crystallinity is observed for the tubes (Figure 2b), CNTs have an amorphous structure, and the microcrystalline structure features many defects.

The synthesized ACNTs had some defects because H_3_PO_4_ strongly reduced the carbon surface, and the produced oxygen might have etched the carbon tube surface into deep pits. The energy dispersive X-ray spectroscopy (HRTEM-EDX) analysis corresponding to the white rectangle area in Figure 2c indicates that the distribution of elements in the ACNTs revealed that carbon, oxygen, and phosphorus were all uniformly distributed. Phosphorus is the acidic catalysts residue (H_3_PO_4_). Its presence confirms that the tubes grew from the furfuryl alcohol to which H_3_PO_4_ was added to trigger its polycondensation. Phosphorus was not present in the carbon background obtained from chitosan by its carbonization prior to saturation with the FA/H_3_PO_4_ mixture. 

**Figure 3:**
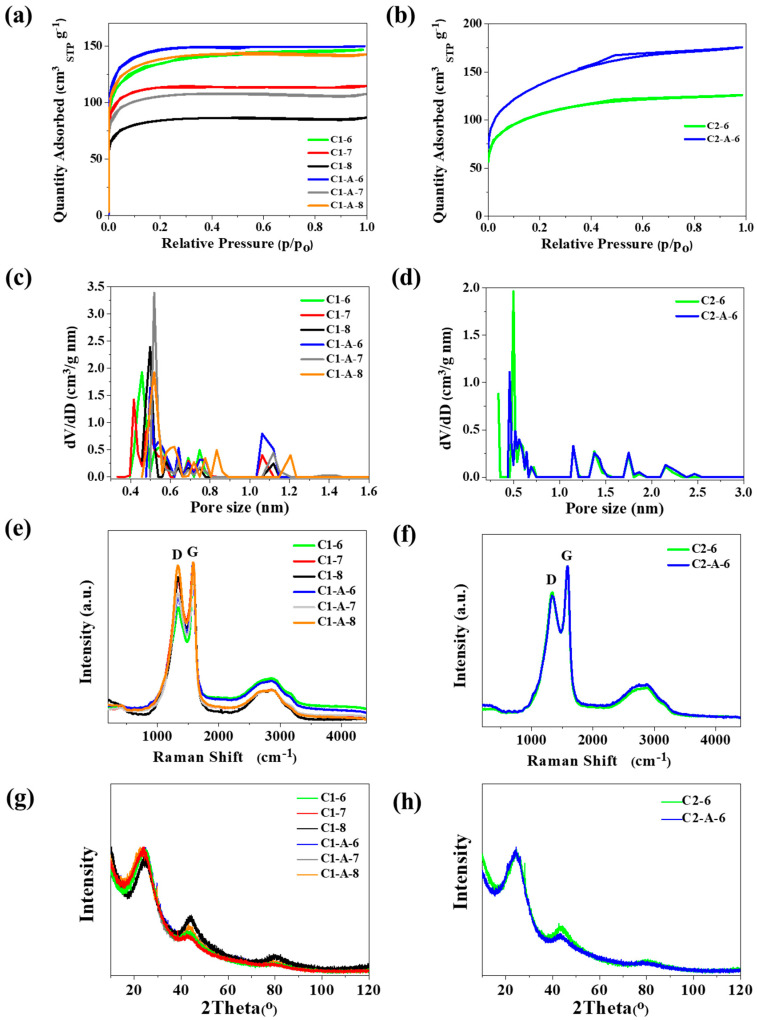
(**a**,**b**) Nitrogen adsorption-desorption isotherms; (**c**,**d**) Pore size distribution by nonlocalized density functional theory (NLDFT) method; (**e**,**f**) Raman spectra for samples C1-A-T, C1-T, C2-A-6, and C2-6; XRD patterns of (**g**) C1-A-T and C1-T; and (**h**), C2-A-6, and C2-6 sample.

The specific surface areas of the ACNTs/N-doped AC hybrid were analyzed using N_2_ gas adsorption and desorption measurements as shown in Figure 3a,b. From these measurements, the specific surface area was calculated using the BET method, where the relative pressure of P/Po is ranged from 0.05 to 0.3. Based on the BET analysis, the specific surface areas of the C1-A-6 and C1-6 samples are 557 and 500 m^2^ g^−1^, respectively. For the ACNTs/N-doped AC hybrid, the nitrogen adsorption–desorption isotherm exhibits an IUPAC type I curve characteristic, indicating that there are many micropores existing in these hybrid materials. The porous property of the ACNTs/N-doped AC hybrid can be further confirmed by the pore size distribution analysis determined by the nonlocalized density functional theory (NLDFT) method. Thanks to this method, we can see that there are micropores respectively centered between 0.4–0.6 nm and 1.0–1.2 nm for series C1 (Figure 3c). For series C2 (Figure 3d) the micropores are respectively centered between 0.5–0.6 nm and 1.0–2.5 nm. The sorption isotherm of the series ACNTs/N-doped AC obtained using C1 N-doped carbon indicates that the morphology and structure is similar to hybrids obtained with C2 N-doped carbon. Elemental CHN analysis was used to further explore the influence of carbonization conditions on elemental content in a ACNTs/N-doped AC hybrid (Table 2). 

The carbon content is high above 70 wt.% for all samples. The nitrogen content ranges between 1.2 and 2.1 wt.%. Table 2 summarizes the specific surface area (S_BET_) for the ACNTs-AC samples. Table 2 provides more information about the average metering nitrogen content in the investigated materials. This parameter is crucial regarding the main aim of the study, which is the synthesis of ACNT-AC hybrids with increased nitrogen content. The N content of 1.8–3.3 wt.%, as presented in Table 2, is much higher than for standard ACs obtained from, for example, plain wood (usually no more than 0.5 wt.%). Thus, the data show that the proposed synthesis concept was worth investigating. The S_BET_ values of hybrid carbon materials are not surprisingly high and are at a level similar for activated carbons. However, it ought to be noted that such S_BET_ values are typical for ACs obtained from chitosan when activation by H_3_PO_4_ and the process were similar to those applied in this study.

Raman spectroscopy is one of the most widely used nondestructive techniques to characterize carbon materials, particularly for distinguishing ordered and disordered crystal structures, defect density, and doping levels of carbon materials. In this study, it was used to characterize the change in the carbon textures after the ACNTs growth at different carbonization temperatures. Raman studies were performed aiming at determining the crystallinity/defect which was not possible in SEM studies. The Raman spectra shown in Figure 3e,f for ACNTs-AC samples display characteristic bands for graphene structures D, G, and 2D. The peak at 1585 cm^−1^ was noted as the G-band which represents the C–C stretching mode of highly ordered graphitic layers with an sp^2^ orbital structure, while the peak at 1340 cm^−1^ was the D-band which mainly corresponds to lattice defects and amorphous carbon. The hump at 2850 cm^−1^ is assigned to the 2D band that is basically an overtone of the D peak involving two in-plane transverse optic phonons. The intensity ratio of I_D_/I_G_ for characteristic bands from the Raman spectra. The G peak is a result of in-plane vibrations of sp^2^ bonded carbon atoms whereas the D peak is due to out of plane vibrations attributed to the presence of structural defects. The I_G_/I_D_ ratio of the ACNTs/N-doped AC is below 1 and it shows that these carbon tubes have many defects in their tube structure. The relative peak ratio intensity of D to G indicates that the templated ACNTs have a lower crystallinity than graphite sheets. Higher pyrolysis temperatures cause an increase in the crystallinity of ACNTs/N-doped AC. This is observed as the ratio of I_D_/I_G_ increases along with the carbonization temperature increasing from 600 to 800 °C. The differences in I_D_/I_G_ ratios confirm that obtained hybrid carbon materials contain different amount of defects in carbon structure. 

The XRD patterns of C1-A-T, C1-T, C2-A-6, and C2-6 are shown in Figure 3g,h. The spectra are characteristic from activated carbons of low crystallinity. Two broad diffraction peaks around 24° and 43° are visible as only peaks in the whole spectra. The peaks correspond to the diffraction of (002) and (100), respectively. The presence of the amorphous carbon nanotubes did not alter the principal character of XRD spectra i.e., no significant differences are visible between the samples. Crystalline nature of CNTs is usually reflected by the sharpening of the peak localized at 26° also corresponding to the diffraction of (002), but the effect is not observable in the presented XRD spectra. The patterns are owing their shape to the dominating presence of activated carbon background, and amorphous CNT definitely present in the investigated materials, did not alter the situation. 

**Figure 4:**
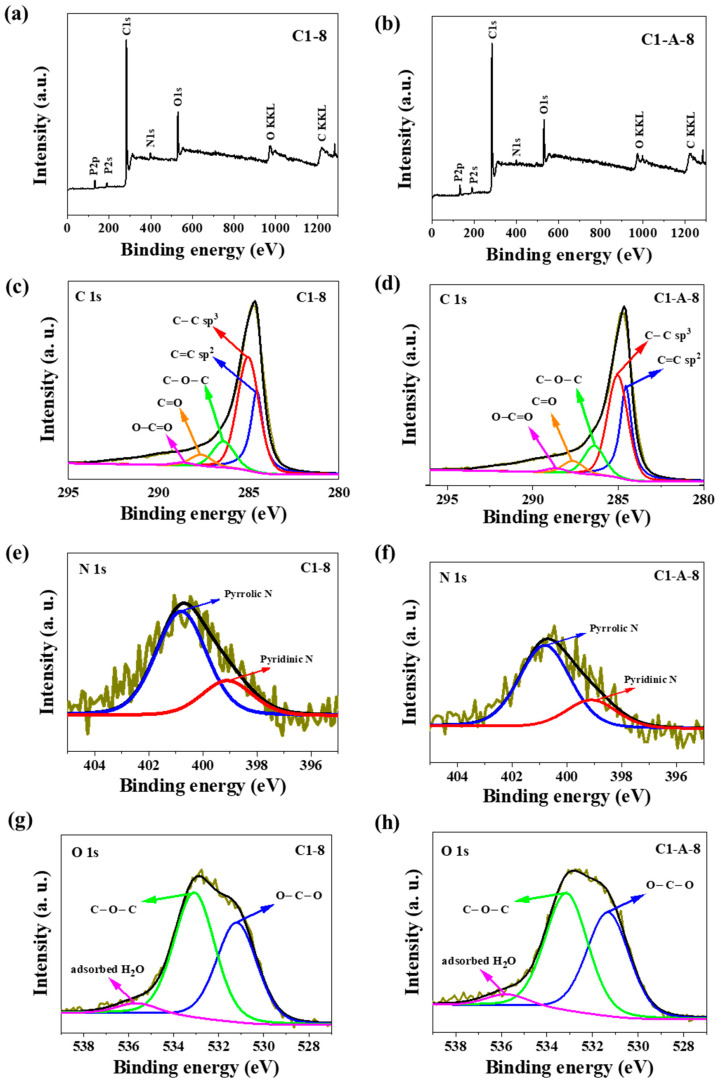
XPS survey spectra (**a**,**b**) and high-resolution XPS spectra for C1s, N1s, and O1s of (**c**,**e**,**g**) C1-8; and (**d**,**f**,**h**) C1-A-8.

The carbon, oxygen, and nitrogen content of ACNT/N-doped AC was tested by the XPS. Figure 4a,b presents survey spectra and high resolution XPS spectra (Figure 4c–h) for all elements detected. Each spectrum was subjected to deconvolution process using PHI Multipak software. The C1s spectra of MWCNTs/N-doped AC are composed of seven peaks corresponding to C=C bond (sp^2^) peak at 284.6 eV [50], C–C bond (sp^3^) peak at 285.0 eV [50,51], C–O–C or C–OH or C–NH bond peak at 286.4 eV [50,51], C=O or O–C–O or N–C–O bond peak at 287.7 eV [50,51], O–C=O peak at 288.6 eV [50,52,53]. The spectra are distinctive because of a high share of aromatic carbon (binding energy of 284.6 eV), i.e., the contribution is ranking from 27.1 to 32.7 at.%. Although the aromatic carbon share is almost as high as that of the C–O–C carbon, the corresponding values are much lower than for typical crystalline CNTs in which the total carbon content may exceed 90 at.% [54]. The fact may be interpreted as a result of: (i) partial crystallinity of the tubes (high share of non-aromatic carbon) and (ii) the presence of an amorphous carbon background obtained by carbonization of chitosan. The first (i) assumption is consistent with SEM and HRTEM observations as well as with the Raman studies. The XPS data prove the presence of nitrogen atoms directly bonded to carbon atoms. The deconvolution of O1s peaks was less informative and revealed only a few specific chemical oxygen states. The peak at 531.6 eV may be interpreted as the presence of O=C–N and/or C=O bonds. The binding energy of 533.2 eV is usually a characteristic of O*=C–O and/or O–C–O bonds. The peak at 536.0 eV indicates the presence of a strongly absorbed H_2_O molecule which has not been desorbed during sample degassing [50,51,52]. The XPS P2p spectrum shows the P–O bond at about 132.9 eV [55]. The high-resolution N1s spectra in the two samples (Figure 4e,f) can be fitted into two N peaks, centered at approximately 399.1 and 400.5 eV, which correspond to pyrrolic-N and quaternary-N, respectively. Table 3 shows the N contents for all samples. The C1-6 and C2-A-6 samples had the highest proportion of pyrrolic-N equal to 2.0 and 2.2 at. %, respectively, and much lower proportion of quaternary-N similar to other samples. However, C2-A-6 had the highest proportion of quaternary-N of all samples and amounted to 1.3 at.%. Figure 4 shows that the N contents of C1-8 and C1-A-8 similarly increased. Such a chemical identity of nitrogen atoms bonded to the surface carbon atoms is very much favored by further electrochemical applications as electrode materials in supercapacitors or as an electrocatalyst for metal–air batteries. On the whole, the chemical composition of nitrogen atoms bonded to a carbon matrix is more important than the total content of nitrogen. In supercapacitor electrodes, the pyrrolic-N generated on the edge sites of the graphitic framework can deliver significantly higher capacitance than the quaternary-N generated on the basal plane. It is due to their ability to contribute to surface faradaic pseudocapacitance in alkaline electrolytes, which leads to enhance the capacitance performance of the electrode [56,57,58].

The presence of the quaternary-N bond can facilitate the electron transfer and cause the enhancement of conductivity in carbon materials. These properties are beneficial for rate and cycling performance of supercapacitors [59,60]. In the case of metal-air batteries, Park et al. concluded that quaternary-N enhanced the limiting current density due to the fact that N atoms reduce the electron density on the adjacent C nuclei, which facilitates electron transfer from the adjacent C to N atoms and N donates electrons back to adjacent C orbitals [46]. Therefore, based on the above experimental results, we believe that the high surface area with quaternary-N groups could be a key factor enhancing potential catalytic activity for oxygen reduction reaction (ORR). Both the unique architecture of quaternary-N groups and the porous structure of ACNT/N-doped AC hybrid carbon could have a synergic effect on highly enhanced ORR activity in metal-free catalysts, because nitrogen groups turned out to be significant active sites along both outer and inner morphology.

## 4. Conclusions

In summary, ACNT/N-doped AC hybrid carbon tubes were prepared by free growth of polymers on a nitrogen-doped carbon surface. The results show that hybrid carbon materials obtained with FeCl_3_ as an additional catalyst cause a higher amount of ACNTs to grow on an N-doped surface. The specific surface area is larger than for samples obtained without FeCl_3_. Tests carried out using a scanning electron microscope (SEM) and a high-resolution transmission electron microscope (HRTEM) will determine the size and shape of the produced amorphous carbon nanotubes. The XRD study confirms the amorphous nature of ACNTs, whereas the SEM and HRTEM studies provide information about the microscopic structures. The ACNTs obtained on the N-doped carbon surface have tube thickness in the range of 40–60 nm. The highest specific surface area was obtained for samples C1-A-6 and C1-6 and it is 557 and 476 m^2^g^−1^, respectively. The obtained hybrid carbon materials of ACNT/N-doped AC will be successfully used in many areas, such as an electrode material for batteries or in supercapacitors.

## Figures and Tables

**Table 1:** Summary of experimental procedure for sample preparation.

Carbon Background	Short Name of Carbon Background	Addition of FeCl_3_	Carbonization Temperature (°C)		
			600	700	800
			Sample Name		
CH_2.46 *	C1	-	C1-6	C1-7	C1-8
CH_5.53 *	C2	-	C2-6	-	-
CH_2.46 *	C1	0.01 g	C1-A-6	C1-A-7	C1-A-8
CH_5.53 *	C2	0.01 g	C2-A-6	-	-

* Carbon obtained from chitosan according to procedure [49].

**Table 2:** Specific surface area and elemental composition by combustion analysis of hybrid carbon materials.

Sample	Content (wt.%)			S_BET_ (m^2^ g^−1^)
	N	C	H	
C1-6	2.6	78.8	1.9	500
C1-7	2.3	68.6	1.7	437
C1-8	2.2	69.8	1.2	320
C1-A-6	3.3	71.2	1.7	557
C1-A-7	2.3	66	1.8	403
C1-A-8	2.2	64.8	1.6	524
C2-6	1.8	78	2	392
C2-A-6	2.1	73.9	2.1	356

**Table 3:** Chemical composition analyzed by X-ray photoelectron spectroscopy (XPS) for C1-T, C1-A-T, C2-T, and C2-A-T series.

Sample	Binding Energy (eV)										
	284.6	285.0	286.4	287.7	288.6	531.6	533.2	536.0	399.1	400.5	132.9
	Elemental Content (at.%)										
	C					O			N		P
C1-6	27.1	38.4	12.3	4.4	2.3	3.9	7.2	0.5	2.0	0.5	1.4
C1-7	30.2	37.0	11.1	3.7	2.0	4.7	6.4	0.5	1.3	0.4	2.6
C1-8	29.5	38.5	8.9	4.0	1.1	5.6	7.0	0.6	1.5	0.5	3.0
C1-A-6	31.1	35.7	9.9	3.9	1.9	6.0	6.8	0.6	1.7	0.6	2.0
C1-A-7	31.9	36.2	9.6	4.0	1.1	5.5	6.4	0.6	1.7	0.5	2.5
C1-A-8	32.7	34.4	9.7	4.2	1.6	5.9	6.7	0.7	1.4	0.5	2.1
C2-6	30.7	39.8	9.2	3.9	1.2	4,8	5.9	0.4	1.6	0.5	2.1
C2-A-6	31.5	33.1	9.3	4.4	2.2	7.6	6.5	0.4	2.2	1.3	1.5
